# Ten-year clinical outcomes of polymer-free versus durable polymer new-generation drug-eluting stent in patients with coronary artery disease with and without diabetes mellitus: Results of the Intracoronary Stenting and Angiographic Results: Test Efficacy of Sirolimus- and Probucol- and Zotarolimus-Eluting Stents (ISAR-TEST 5) trial

**DOI:** 10.1007/s00392-021-01854-7

**Published:** 2021-06-22

**Authors:** Tobias Koch, Tobias Lenz, Michael Joner, Erion Xhepa, Tobias Koppara, Jens Wiebe, J. J. Coughlan, Alp Aytekin, Tareq Ibrahim, Thorsten Kessler, Salvatore Cassese, Karl-Ludwig Laugwitz, Heribert Schunkert, Adnan Kastrati, Sebastian Kufner, for the Intracoronary Stenting and Angiographic Results: Test Efficacy of Sirolimus- and Probucol-Eluting Versus Zotarolimus- Eluting Stents (ISAR-TEST 5) Investigators, Julinda Mehilli, Jörg Hausleiter, Adnan Kastrati, Robert A. Byrne, Tareq Ibrahim, Karl-Ludwig Laugwitz, Heribert Schunkert, Sebastian Kufner

**Affiliations:** 1 Deutsches Herzzentrum München, Klinik Für Herz- Und Kreislauferkrankungen, an der Technischen Universität München, Lazarettstrasse 36, 80636 Munich, Germany; 2 1. medizinische Klinik, Klinikum Rechts der Isar, Technische Universität, Munich, Germany; 3 DZHK (German Centre for Cardiovascular Research), Partner Site Munich Heart Alliance, Munich, Germany

**Keywords:** Drug-eluting stent, Durable polymer, Long-term follow-up, Polymer free, Probucol, Randomized controlled trial, Sirolimus, Zotarolimus, Diabetes mellitus

## Abstract

**Background:**

Very long-term outcomes according to diabetic status of patients with coronary artery disease (CAD) undergoing percutaneous coronary intervention (PCI) with new-generation drug-eluting stents (DES) are scant. Both, the durable polymer zotarolimus-eluting stent (DP-ZES), the first DES to gain FDA-approval for specific use in patients with diabetes mellitus, and the polymer-free sirolimus- and probucol-eluting stent (PF-SES), with a unique design that enables effective drug release without the need of a polymer offer the potential to enhance clinical long-term outcomes especially in patients with diabetes mellitus.

**Methods:**

We investigate 10-year clinical outcomes of the prespecified subgroups of patients with and without diabetes mellitus, randomly assigned to treatment with PF-SES versus DP-ZES in the ISAR-TEST 5 trial. The primary endpoint of interest was major adverse cardiac events (MACE), defined as the composite of all-cause death, any myocardial infarction or any revascularization. Further endpoints of interest were cardiac death, myocardial infarction related to the target vessel and target lesion revascularization as well as the individual components of the primary composite endpoint and the incidence of definite or probable stent thrombosis at 10 years.

**Results:**

This analysis includes a total of 3002 patients randomly assigned to PF-SES (*n* = 2002) or DP-ZES (*n* = 1000). Prevalence of diabetes mellitus was high and comparable, 575 Patients (28.7%) in PF-SES group and 295 patients (29.5%) in DP-ZES group (*P* = 0.66). At 10 years 53.5% of patients with diabetes mellitus and 68.5% of patients without diabetes mellitus were alive. Regarding major adverse cardiac events, PF-SES as compared to DP-ZES showed comparable event rates in patients with diabetes mellitus (74.8% vs. 79.6%; hazard ratio 0.86; 95% CI 0.73–1.02; *P* = 0.08) and in patients without diabetes (PF-SES 62.5% vs. DP-ZES 62.2%; hazard ratio 0.99; 95% CI 0.88–1.11; *P* = 0.88).

**Conclusion:**

At 10 years, both new-generation DES show comparable clinical outcome irrespective of diabetic status or polymer strategy. Event rates after PCI in patients with diabetes mellitus are considerable higher than in patients without diabetes mellitus and continue to accrue over time.

**Trial registration:**

ClinicalTrials.gov, NCT00598533, Registered 10 January 2008, https://clinicaltrials.gov/ct2/show/NCT00598533?term=NCT00598533

**Graphic abstract:**

Kaplan-Meier estimates of endpoints of interest for patients with vs. without diabetes mellitus treated with PF-SES vs. DP-ZES. Bar graphs: Kaplan-Meier estimates as percentages. PF-SES: polymer-free sirolimus-eluting stent; DP-ZES: durable polymer zotarolimus-eluting stent; DM: diabetes mellitus. Comparison of event rates of individual endpoints in patients with and without diabetes mellitus treated with PF-SES vs. DP-ZES all without statistically significant differences. Comparison of event rates of individual endpoints in overall patients with vs. without diabetes mellitus significantly different (*P* ≤ 0.01 for all comparisons).
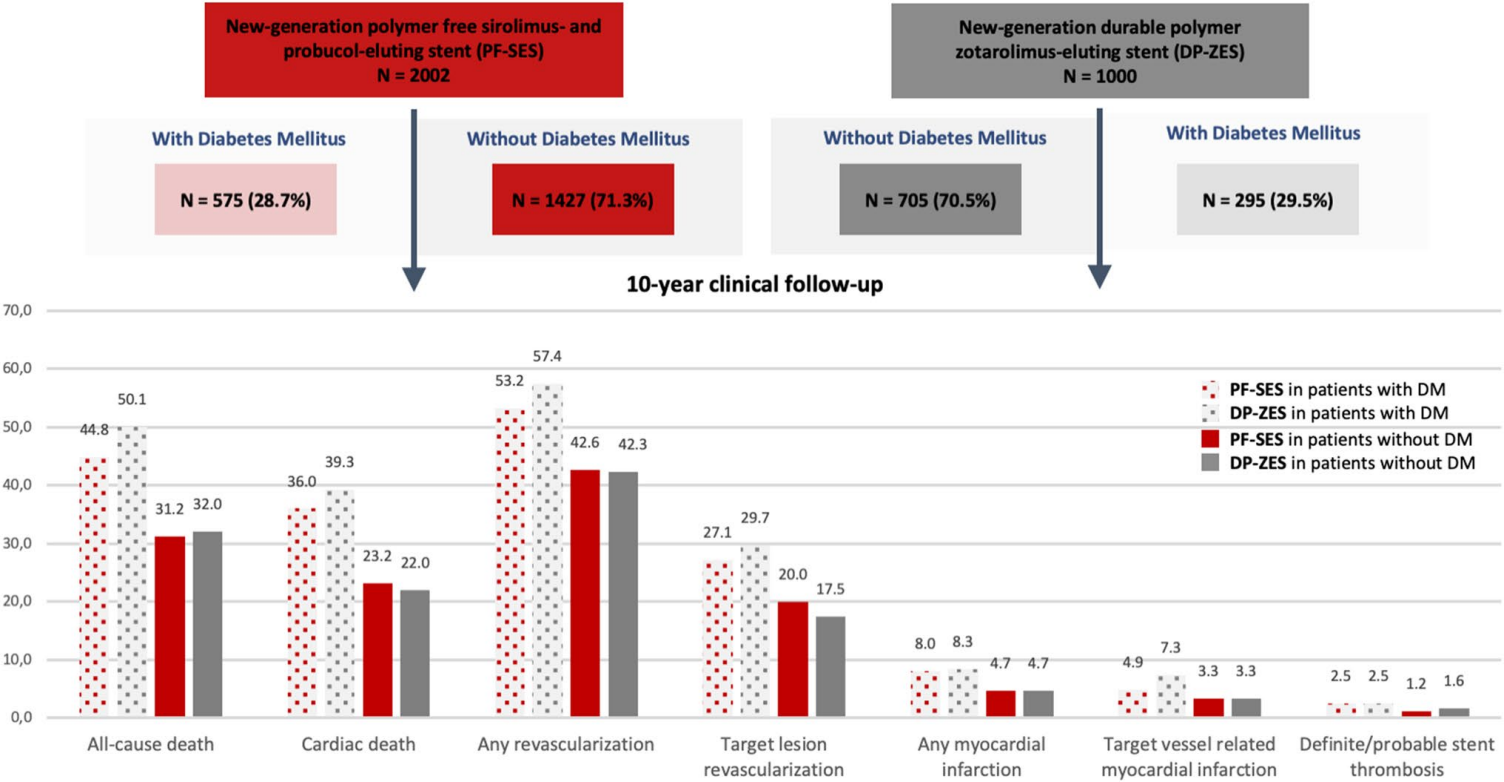

**Supplementary Information:**

The online version contains supplementary material available at 10.1007/s00392-021-01854-7.

## Background

Diabetes mellitus is associated with numerous acute and late complications affecting different organ systems. However, cardiovascular disease remains the leading cause of morbidity and mortality in this population. In this vein, myocardial revascularization strategies remain a crucial part of the treatment of these patients [1, 2]. While current evidence favors coronary artery bypass grafting as the treatment of choice in patients with diabetes and complex multivessel disease, the growing number of patients treated with percutaneous coronary intervention (PCI) and drug-eluting stent (DES) implantation in complex disease including left main stenosis and patients with increased surgical risk remains considerable [2, 3].

Since atherosclerotic lesions in patients with diabetes mellitus are known to present greater inflammation than in other patients [4], device innovations specifically address the drug-carrying polymer attempting to reduce polymer-induced inflammatory stimuli, as had been revealed by pathology studies [5, 6]. Different approaches to meet this issue included permanent polymers with improved biocompatibility and polymer-free DES. In this vein, the first device to gain FDA approval for specific use in diabetic patients was the zotarolimus-eluting stent, based on a specific durable polymer with higher biocompatibility (DP-ZES) [7]. On the other hand, the polymer-free sirolimus- and probucol-eluting stent (PF-SES) is a DES with a unique design that enables effective drug release without the need of a polymer. Although the effects of which are believed to become evident over time, very long-term outcomes of diabetic patients treated with either of these DES beyond 5-year follow-up have not been assessed to date.

In this context, we report 10-year clinical outcomes of the prespecified subgroups of patients with and without diabetes mellitus, enrolled in the ISAR-TEST 5 randomized controlled trial to compare a polymer-free probucol- and sirolimus-eluting stent versus a new-generation durable polymer zotarolimus-eluting stent in coronary artery disease.

## Methods

### Study population, device description and study protocol

The primary analysis, including full details of the study population, methods and endpoints, of the ISAR-Test 5 trial was previously reported [8]. Patients with diabetes mellitus represented a prespecified subgroup of interest according to the trial protocol. In brief, ISAR-Test 5 was a randomized controlled trial, that enrolled patients older than 18 years of age with ischaemic symptoms or evidence of myocardial ischaemia (inducible or spontaneous) in the presence of written, informed consent by the patient or her/his legally authorized representative for participation in the study was obtained. Patients with a target lesion located in the left main stem, cardiogenic shock, malignancies or other co-morbid conditions with life expectancy less than 12 months or that may result in protocol non-compliance, known allergy to the study medications (probucol, sirolimus, zotarolimus) or pregnancy (present, suspected or planned) were considered ineligible for the study. The trial protocol was approved by the institutional ethics committee of the two participating centers: Deutsches Herzzentrum München and 1. Medizinische Klinik, Klinikum Rechts der Isar, both in Munich, Germany.

Patients who met all of the inclusion criteria and none of the exclusion criteria were randomized in the order that they qualified. Patients were assigned to receive polymer-free sirolimus- and probucol-eluting stents or durable polymer zotarolimus-eluting stents in a 2:1 allocation. The polymer-free sirolimus- and probucol-eluting stents consists of a pre-mounted, sand-blasted, thin-strut 316L stainless steel microporous stent which is coated with a mixture of sirolimus, probucol, and shellac resin (a biocompatible resin widely used in the coating of medical tablets). (This coating strategy is currently available in two devices: ISAR VIVO, Translumina Therapeutics, Dehradoon, India, Translumina, Hechingen, Germany and Coroflex ISAR, B. Braun Melsungen, Berlin, Germany.) The durable polymer zotarolimus-eluting stent (Resolute, Medtronic Cardiovascular, Santa Rosa, CA) consists of a thin-strut 91-µm stent platform. The polymer-coating system consists of three different polymers: a hydrophobic C10 polymer, a hydrophilic C19 polymer and polyvinylpyrrolidinone. Further detailed descriptions of stent platforms and elution characteristics of both stents have been reported previously [9–12]. The aim of the current study was to compare outcomes of patients treated with polymer-free sirolimus- and probucol-eluting stents versus durable polymer zotarolimus-eluting stent after extended clinical follow-up out to 10 years.

### End points, and definitions

The primary endpoint of the present analysis was the composite of all-cause death, any myocardial infarction or any revascularization (major adverse cardiac events; MACE). Further endpoints of interest were cardiac death, myocardial infarction related to the target vessel and target lesion revascularization at 10 years, as well as the individual components of the primary composite endpoint and the incidence of definite or probable stent thrombosis (by Academic Research Consortium definition) at 10 years. Detailed description of study endpoints and definitions have also been reported previously [8].

### Follow-up and analysis

Patients were systematically evaluated at 1 and 12 months and annually out to 10 years. Extended follow-up was performed in the setting of routine care by either telephone calls or office visit in the two participating centers. The study was conducted in accordance with the provisions of the Declaration of Helsinki and with the International Conference on Harmonization Good Clinical Practices. All patients provided written informed consent for participation in the clinical trial. Analysis of data from extended follow-up, which was not prespecified in the trial protocol, was approved by the institutional ethics committee responsible for the participating centers. Additional written informed consent from patients was waived. All events were adjudicated and classified by an event adjudication committee blinded to treatment allocation.

### Statistical analysis

Continuous data are presented as mean (standard deviation) or median [25th–75th percentiles]. Categorical data are presented as counts or proportions (%). Data distribution was tested for normality using the Kolmogorov–Smirnov test for goodness of fit. For patient-level data, differences between groups were checked for significance using Student’s *t* test or Wilcoxon rank sum test (continuous data) or the chi-squared or Fisher’s exact test where the expected cell value was < 5 (categorical variables). For lesion level data, differences between groups were checked for significance using generalized estimating equations for non-normally distributed data to address intra-patient correlation in patients who underwent multi-lesion intervention [13].

Event-free survival was assessed using the methods of Kaplan–Meier. Hazard ratios, confidence intervals and p values were calculated from univariate Cox proportional hazards models. The proportional hazards assumption was checked by the method of Grambsch and Therneau [14] and was fulfilled in all cases in which we used Cox proportional hazards models. The analysis of all endpoints was planned to be performed on an intention-to-treat basis [15]. Statistical software R, version 3.6.1 (R Foundation for Statistical Computing, Vienna, Austria) was used for analysis.

## Results

This analysis includes a total of 3002 patients with coronary artery disease randomized to treatment with either polymer-free sirolimus- and probucol-eluting stents (PF-SES: *n* = 2002) or durable zotarolimus-eluting stents (DP-ZES: *n* = 1000) in the setting of the randomized ISAR-TEST 5 trial.

Prevalence of diabetes mellitus was high, 870 patients (29.0%), and comparable in both treatment groups. 575 Patients (28.7%) who received PF-SES and 295 patients (29.5%) who received DP-ZES had diabetes (*P* = 0.66). Over the course of 10-year clinical follow-up, 67 patients treated with PF-SES (4.7%) and 22 patients treated with DP-ZES (3.12%) were newly diagnosed with diabetes mellitus (*P* = 0.111). Overall baseline characteristics according to diabetic status are summarized in Supplemental Table 1.

Baseline characteristics according to diabetic status and treatment group are summarized in Table 1. Baseline patient and lesion characteristics were well balanced between both treatment groups, except one: patients without diabetes mellitus and treated with DP-ZES had significantly more often hyperlipidemia than those who received PF-SES (65.5% vs. 60.8%, *P* = 0.04). 10-year clinical follow-up was completed in 85.1% of the study population, follow-up details have been previously described in detail [16]**.**

**Table 1:** Baseline patient and lesion characteristics in patient with and without diabetes mellitus by treatment group

Characteristics	Patients with diabetes mellitus			Patients without diabetes mellitus		
	PF-SES N = 575	DP-ZES N = 295	*P*	PF-SES N = 1427	DP-ZES N = 705	*P*
Patients						
Age, y, ± SD	68.3 (± 10.2)	69.0 (± 9.7)	0.37	67.4 (± 11.6)	67.8 (± 11.2)	0.50
Male sex	425 (73.9)	216 (73.2)	0.89	1107 (77.6)	547 (77.6)	> 0.99
Insulin-dependent diabetes	197 (34.3)	109 (36.9)	0.48			
Oral antidiabetic medication	289 (50.3)	149 (50.5)	> 0.99			
Arterial hypertension	427 (74.3)	210 (71.2)	0.37	909 (63.7)	456 (64.7)	0.69
Current smoker	105 (18.3)	52 (17.6)	0.89	252 (17.7)	114 (16.2)	0.43
Hyperlipidemia	389 (67.7)	188 (63.7)	0.28	868 (60.8)	462 (65.5)	0.04
Coronary artery disease			0.66			0.08
1-vessel disease	58 (10.1)	32 (10.8)		286 (20.0)	113 (16.0)	
2-vessel disease	130 (22.6)	59 (20.0)		383 (26.8)	199 (28.2)	
3-vessel disease	387 (67.3)	204 (69.2)		758 (53.1)	393 (55.7)	
Clinical presentation			0.46			0.91
Unstable Angina	98 (17.0)	61 (20.7)		267 (18.7)	139 (19.7)	
Non-ST-segment elevation acute coronary syndrome	73 (12.7)	45 (15.3)		158 (11.1)	80 (11.3)	
Silent Ischemia	36 (6.3)	15 (5.1)		100 (7.0)	52 (7.4)	
Stable angina	324 (56.3)	154 (52.2)		731 (51.2)	358 (50.8)	
ST-segment elevation myocardial infarction	44 (7.7)	20 (6.8)		171 (12.0)	76 (10.8)	
Prior myocardial infarction	177 (30.1)	85 (28.8)	0.60	409 (28.7)	214 (30.4)	0.45
Prior coronary artery bypass grafting	59 (10.3)	34 (11.5)	0.65	129 (9.0)	62 (8.8)	0.92
Body Mass Index, ± SD	29.3 (± 4.9)	28.9 (± 4.7)	0.18	27.2 (± 4.4)	26.9 (± 4.1)	0.09
Ejection fraction, %, ± SD	50.9 (± 12.3)	51.1 (± 12.7)	0.84	53.2 (± 11.6)	52.9 (± 10.8)	0.60
Lesions						
Vessel			0.77			0.06
LAD	237 (41.2)	129 (43.7)		684 (47.9)	301 (42.7)	
LCx	161 (28.0)	78 (26.4)		334 (23.4)	189 (26.8)	
RCA	177 (30.8)	88 (29.8)		409 (28.7)	215 (30.5)	
Ostial	93 (16.2)	51 (17.3)	0.75	256 (17.9)	133 (18.9)	0.65
Bifurcational	115 (20.0)	60 (20.3)	0.98	334 (23.4)	192 (27.2)	0.06
Chronic occlusion	33 (5.8)	20 (6.8)	0.65	74 (5.2)	39 (5.5)	0.82

Data shown as means (± SD) or number (percentage)

### Clinical outcomes of PF-SES versus DP-ZES in patients with and without diabetes mellitus at 10 years

Clinical results according to diabetic status (patients without and with diabetes mellitus) are summarized in Supplemental Table 2. Clinical results according to diabetic status and treatment group are summarized in Table 2.

**Table 2:** Clinical outcomes at 10 years in patients with and without diabetes mellitus, hazard ratios, by treatment group

	Patients with diabetes mellitus				Patients without diabetes mellitus			
	PF-SES *N* = 575	HR (95% CI) PF-SES versus DP-ZES	DP-ZES *N* = 295	*P*	PF-SES *N* = 1427	HR (95% CI) PF-SES versus DP-ZES	DP-ZES *N* = 705	*P*
MACE	403 (74.8)	0.86 (0.73–1.02)	225 (79.6)	0.08	855 (62.5)	0.99 (0.88–1.11)	420 (62.2)	0.88
All-cause death	228 (44.8)	0.84 (0.68–1.04)	135 (50.1)	0.11	409 (31.2)	0.96 (0.81–1.13)	208 (32.0)	0.60
Any myocardial infarction	42 (8.0)	0.97 (0.58–1.63)	22 (8.3)	0.92	61 (4.7)	1.00 (0.64–1.54)	30 (4.7)	0.99
Any revascularization	266 (53.2)	0.92 (0.75–1.13)	140 (57.4)	0.43	554 (42.6)	1.00 (0.90–1.16)	269 (42.3)	0.97
Cardiac death	163 (36.0)	0.89 (0.69–1.15)	91 (39.3)	0.38	275 (23.2)	1.06 (0.86–1.31)	126 (22.0)	0.60
Target vessel related myocardial infarction	27 (4.9)	0.72 (0.40–1.30)	19 (7.3)	0.27	42 (3.3)	0.94 (0.56–1.57)	22 (3.3)	0.80
TLR	126 (27.1)	0.95 (0.71–1.28)	66 (29.7)	0.75	245 (20.0)	1.10 (0.87–1.37)	109 (17.5)	0.43

Data are shown as number (Kaplan–Meier estimates as percentages), hazard ratios are derived from Cox proportional hazard models, and P values are derived from Cox proportional hazard models. PF-SES indicates biodegradable polymer-free sirolimus- and probucol-eluting stent; DP-ZES indicates durable polymer zotarolimus- eluting stent, MACE = major adverse cardiac events, defined as the composite of all-cause death, any myocardial infarction and any revascularization

**Fig. 1:**
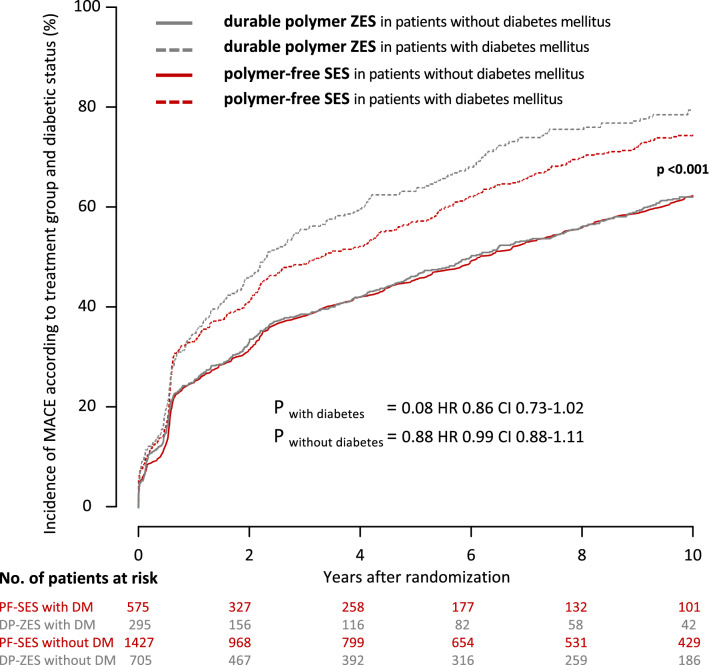
Kaplan–Meier curves for incidence of major adverse cardiac events according to treatment group and diabetic status. *PF-SES* polymer-free sirolimus-eluting stent, *DP-ZES* durable polymer zotarolimus-eluting stent, *DM* diabetes mellitus, *MACE* major adverse cardiac events, *HR* hazard ratios derived from Cox proportional hazard models, *CI* confidence interval, *P*_overall with vs. without DM_ indicates the overall comparison of patients with diabetes versus patients without diabetes irrespective of stent type

Concerning the composite of all-cause death, any myocardial infarction and any revascularization, rates were high but comparable in patients with diabetes mellitus treated with PF-SES as compared to DP-ZES (74.8% vs. 79.6%; *P* = 0.08; hazard ratio 0.86; 95% CI 0.73–1.02) and patients without diabetes mellitus (PF-SES 62.5% vs. DP-ZES 62.2%; *P* = 0.88; hazard ratio 0.99; 95% CI 0.88–1.11). Kaplan–Meier curves for the incidence of major adverse cardiac events according to treatment group and diabetic status are displayed in Fig. 1.

**Fig. 2:**
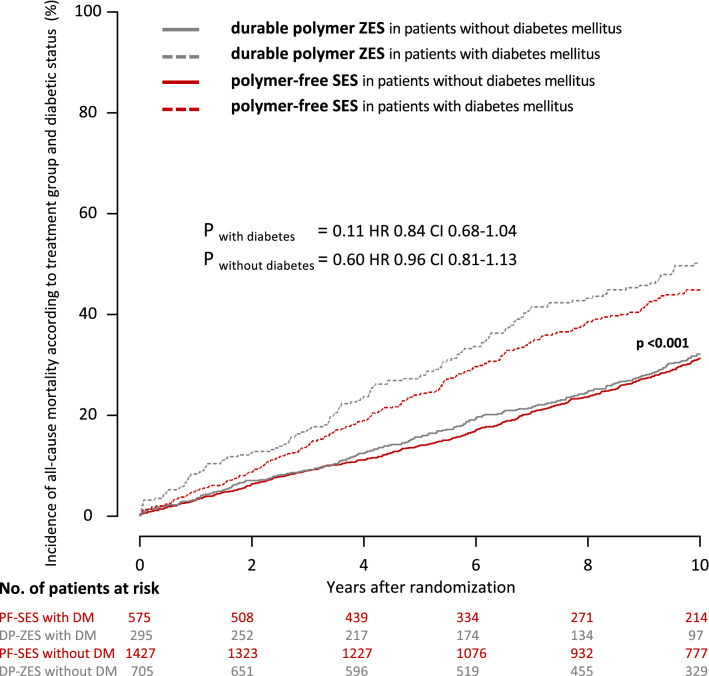
Kaplan–Meier curves for incidence of all-cause death according to treatment group and diabetic status. *PF-SES* polymer-free sirolimus-eluting stent, *DP-ZES* durable polymer zotarolimus-eluting stent, *DM* diabetes mellitus, *HR* hazard ratios derived from Cox proportional hazard models, *CI* confidence interval, *P*_*overall with vs. without DM*_ indicates the overall comparison of patients with diabetes versus patients without diabetes irrespective of stent type

At 10 years, 53.5% of patients with diabetes mellitus and 68.5% of patients without diabetes mellitus were alive. All-cause mortality rates were comparable in patients with diabetes mellitus treated with PF-SES as compared to DP-ZES (44.8% vs. 50.1%; *P* = 0.11; hazard ratio 0.84; 95% CI 0.68–1.04) and patients without diabetes mellitus (PF-SES 31.2% vs. DP-ZES 32.0%; *P* = 0.60; hazard ratio 0.96; 95% CI 0.81–1.13). Kaplan–Meier curves for the incidence of all-cause death according to treatment group and diabetic status are displayed in Fig. 2.

**Fig. 3:**
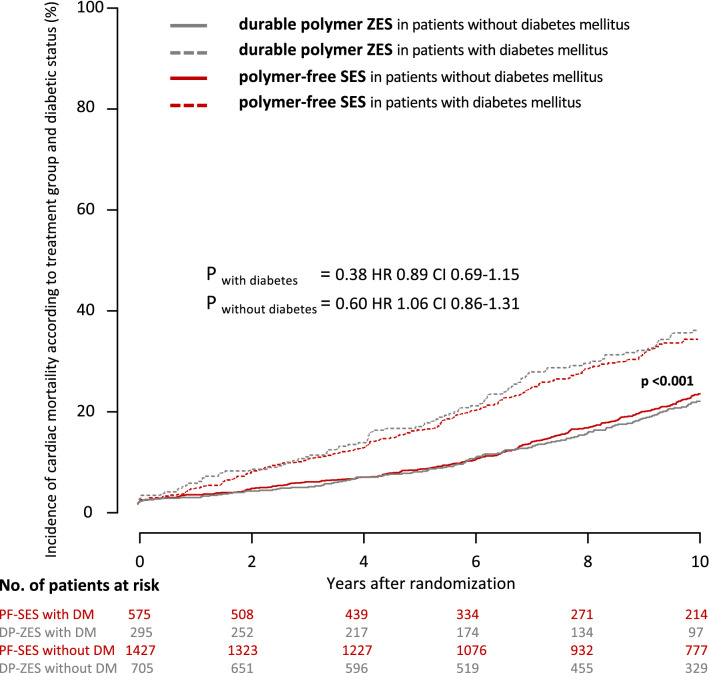
Kaplan–Meier curves for incidence of cardiac mortality according to treatment group and diabetic status. *PF-SES* polymer-free sirolimus-eluting stent, *DP-ZES* durable polymer zotarolimus-eluting stent, *DM* diabetes mellitus, *HR* hazard ratios derived from Cox proportional hazard models, *CI* confidence interval, *P*_*overall with vs. without DM*_ indicates the overall comparison of patients with diabetes versus patients without diabetes irrespective of stent type

Rates of cardiac death at 10 years were comparable between PF-SES and DP-ZES in patients with diabetes (36.0% vs. 39.3%; *P* = 0.38; hazard ratio 0.89; 95% CI 0.69–1.15). Patients without diabetes had overall lower rates of cardiac death, but without any significant difference between treatment groups (PF-SES 23.2% vs. DP-ZES 22.0%; *P* = 0.60; hazard ratio 1.06; 95% CI 0.86–1.31). Kaplan–Meier curves for the incidence of cardiac mortality according to treatment group and diabetic status are displayed in Fig. 3.

Regarding the incidence of any myocardial infarction at 10 years, there was no significant difference between PF-SES and DP-ZES in patients with diabetes mellitus (PF-SES 8.0% vs. DP-ZES 8.3%; *P* = 0.92, hazard ratio 0.97; 95% CI 0.58–1.63) and patients without diabetes mellitus (PF-SES 4.7% vs. DP-ZES 4.7%, *P* = 0.99, hazard ratio 1.00; 95% CI 0.64–1.54).

Regarding the incidence of target vessel related myocardial infarction at 10 years there was no significant difference between PF-SES and DP-ZES in patients with diabetes (PF-SES 4.9% vs. DP-ZES 7.3%, *P* = 0.27; hazard ratio 0.72; 95% CI 0.40–1.30) and without diabetes (PF-SES 3.3% vs. DP-ZES 3.3%, *P* = 0.80; hazard ratio 0.94; 95% CI 0.56–1.57).

**Fig. 4:**
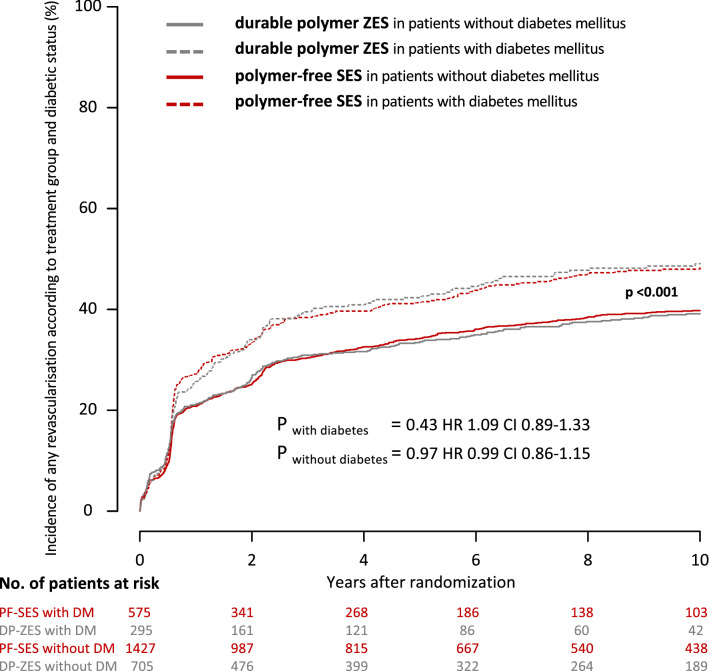
Kaplan–Meier curves for incidence of any revascularization according to treatment group and diabetic status. *PF-SES* polymer-free sirolimus-eluting stent, *DP-ZES* durable polymer zotarolimus-eluting stent, *DM* diabetes mellitus, *HR* hazard ratios derived from Cox proportional hazard models, *CI* confidence interval, *P*_*overall with vs. without DM*_ indicates the overall comparison of patients with diabetes versus patients without diabetes irrespective of stent type

Rates for any revascularization were high but comparable in patients with diabetes mellitus treated with PF-SES as compared to DP-ZES (PF-SES 53.2% vs. DP-ZES 57.4%; *P* = 0.43, hazard ratio 0.92; 95% CI 0.75–1.13) and patients without diabetes mellitus (PF-SES 42.6% vs. 42.3%; *P* = 0.97, hazard ratio 1.00; 95% CI 0.90–1.16). Kaplan–Meier curves for the incidence of any revascularization according to treatment group and diabetic status are displayed in Fig. 4.

**Fig. 5:**
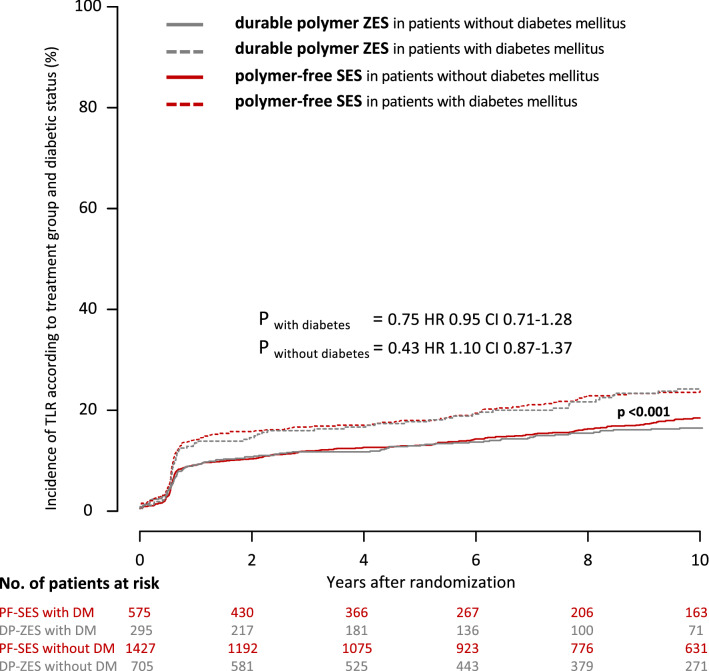
Kaplan–Meier curves for incidence of target lesion revascularization according to treatment group and diabetic status. *PF-SES* polymer-free sirolimus-eluting stent, *DP-ZES* durable polymer zotarolimus-eluting stent, *DM* diabetes mellitus, *HR* hazard ratios derived from Cox proportional hazard models, *CI* confidence interval, *P*_*overall with vs. without DM*_ indicates the overall comparison of patients with diabetes versus patients without diabetes irrespective of stent type

Regarding the incidence of target lesion revascularization in patients with diabetes, rates were comparable in both treatment groups (PF-SES 27.1% vs. DP-ZES 29.7%; *P* = 0.75; hazard ratio, 0.95; 95% CI 0.71–1.28). In patients without diabetes, rates were comparable in both treatment groups (PF-SES 20.0% vs. DP-ZES 17.5%; *P* = 0.43; hazard ratio 1.10; 95% CI 0.87–1.37). Kaplan–Meier curves for the incidence of target lesion revascularization according to treatment group and diabetic status are displayed in Fig. 5.

### Safety outcomes

Regarding safety outcomes, rates of definite/probable stent thrombosis were low and comparable in patients with diabetes mellitus treated with PF-SES as compared to DP-ZES (PF-SES 2.5% vs. DP-ZES 2.5%; *P* = 0.97, hazard ratio 1.02; 95% CI 0.41–2.52) and patients without diabetes mellitus (PF-SES 1.2% vs. DP-ZES 1.6%; *P* = 0.45, hazard ratio 0.74; 95% CI 0.33–1.64). Detailed results concerning incidence of definite, probable stent thrombosis according to diabetic status and treatment group are displayed in Table 3. Results concerning incidence of definite, probable stent thrombosis according to diabetic status are displayed in Supplemental Table 3.

**Table 3:** Definite probable stent thrombosis at 10 years in patients with and without diabetes mellitus

Event	PF-SES	DP-ZES	Hazard ratio	*P*
**With diabetes**	*n* = 575	*n* = 295		
Definite stent thrombosis	7 (1.2)	4 (1.5)	0.89 (0.26–3.04)	0.85
Probable stent thrombosis	7 (1.2)	3 (1.0)	1.19 (0.31–4.60)	0.80
Definite/probable stent thrombosis	14 (2.5)	7 (2.5)	1.02 (0.41–2.52)	0.97
**Without diabetes**	*n* = 1427	*n* = 705		
Definite stent thrombosis	8 (0.6)	3 (0.5)	1.31 (0.35–4.92)	0.69
Probable stent thrombosis	7 (0.5)	7 (1.1)	0.49 (0.17–1.40)	0.18
Definite/probable stent thrombosis	15 (1.2)	10 (1.6)	0.74 (0.33–1.64)	0.45

Data are shown as number (Kaplan–Meier estimates as percentages), hazard ratios are derived from Cox proportional hazard models, and P values are derived from Cox proportional hazard models. PF-SES indicates biodegradable polymer-free sirolimus- and probucol-eluting stent; DP-ZES indicates durable polymer zotarolimus-eluting stent

## Discussion

The present analysis represents a valuable addition to a limited data-set of extended long-term clinical outcome comparisons of new-generation DES and the first report of 10-year clinical outcomes of both: the durable polymer zotarolimus-eluting stents as well as the polymer-free sirolimus- and probucol-eluting stent in patients with and without diabetes mellitus. The main findings of the present study are: first, at 10 years, there was no significant difference in the incidence of both device- and patient-oriented endpoints between patients treated with DP-ZES versus PF-SES, neither in the subgroup of patients with diabetes mellitus nor in the subgroup of patients without diabetes mellitus. Second, irrespective of stent type, overall clinical event rates were considerably worse in patients with diabetes mellitus as compared to patients without diabetes mellitus.

### Long-term follow-up in current DES trials

In recent years, increasing consideration has been given to the long-term outcomes (> 5 years) post-PCI [16, 17]. This represents an important shift in focus to the safety and efficacy of these devices over the lifespan of the patient. This might be of particular importance in patient subgroups with persistent higher event rates over time**,** such as patients with diabetes mellitus. However, traditionally, stent trials have focused on shorter term outcomes, and therefore, current data including this specific high-risk subgroup of patients are limited [18, 19]. Two considerations should be taken into account. First, it has been suggested that the benefit of enhanced polymer strategies, may emerge over time [17]. Second, iterations in stent design aiming on a reduction of persistent inflammatory stimulus caused by permanent polymers might be most beneficial in patients with diabetes mellitus, given the proinflammatory baseline environment in these patients [4].

### New-generation DES and thrombotic events

After preclinical research had revealed that durable polymer might be associated with impaired vascular healing after stent implantation and, therefore, potentially increase the risk for late thrombotic events [5, 6], trials began to evaluate alternative polymer-based and non-polymer-based drug-elution strategies. Different new-generation stent types emerged from these efforts, including polymer-free DES [20]. One promising group of patients in which polymer-free DES are currently being investigated are patients with diabetes mellitus [18, 21, 22]. The low incidence of thrombotic events at 10 years, in this study, with either new-generation DES, PF-SES or DP-ZES is reassuring, and underlines that new-generation DES might have overcome one major drawback of early-generation permanent-polymer DES. This is particularly true concerning late stent thrombosis, with only one event in the overall cohort beyond 12 months. On the other hand, the two-fold higher rates of stent thrombosis in patients with diabetes mellitus at 10 years as compared to patients without diabetes mellitus is noteworthy. Along with these results, the rates of myocardial infarction in this analysis deserve further attention. Interestingly, while target vessel MI rates at 10 years remain two-fold higher in patients with—as compared to patients without—diabetes mellitus, overall event-rates beyond 5 years remain negligible. In contrast, any myocardial infarction continues to occur constantly out to 10 years to 8.1% of patients with diabetes mellitus as compared to 4.7% in non-diabetic patients (*P* < 0.001). This underlines the importance of specific considerations concerning concomitant antithrombotic treatment regimes in patients with diabetes mellitus [23].

### Clinical outcomes at 10 years

Concerning clinical outcomes, in this study, PF-SES has demonstrated comparable but not superior long-term outcomes as compared to new-generation durable polymer ZES. Although, these results are broadly in line with previous results at 5 years in a dedicated analysis of patients with diabetes mellitus and the 10-year results of the overall cohort [16, 18], the cumulative 10-year event rate of almost 80% in patients with diabetes mellitus remains alarming. Therefore, some findings concerning the individual endpoints of interest beyond 5 years deserve further consideration. First, in this analysis, irrespective of diabetic status, rather patient-oriented endpoints—such as any revascularization and all-cause mortality—predominate over rather device-specific endpoints. Accordingly, rates of any revascularization are two-fold higher than target lesion revascularization rates at 10 years in both, patients with and without diabetes mellitus. Additionally, diabetes mellitus is associated with a significant increased 38% relative risk of any revascularization. Both findings are in line with previous observations [24], and underline that disease progression in other coronary segments has greater impact on late clinical outcomes than recurrent events in the intervened lesion. Our data suggest, that this seems specifically true for patients with diabetes mellitus potentially due to a more defuse type of CAD. Concerning mortality, unsurprisingly both cardiac and all-cause mortality was higher in patients with diabetes mellitus as compared to patients without diabetes. Noteworthy, the majority of diabetic patients (70%) died from cardiac cause. Although, these findings contradict to previous registry-based long-term data reporting, that mortality, beyond 1 year after PCI, is mainly driven by non-cardiac death [25], these results underline that cardiovascular disease remains the leading cause of morbidity and mortality in diabetic patients with CAD.

### Polymer-free DES in diabetic patients

Besides the PF-SES investigated in the present study, data from randomized trials and large multicenter registries are available for two further new-generation devices: the polymer-free amphilimus-eluting (PF-AES) and biolimus-eluting (PF-BES) stents, although with follow-up duration not longer than 5 years. In patients with diabetes mellitus, the PF-BES showed superior efficacy and comparable safety over bare-metal stent in the respective subgroup analysis of the LEADERS FREE trial [21]. However, PF-BES failed to meet criteria for non-inferiority when compared to a new-generation ultrathin-strut biodegradable polymer sirolimus-eluting stent in the all-comer SORT-OUT IX randomized trial [26]. The longest term data for PF-AES also derive from the respective first in man trial. The NEXT trial randomized selected patients to treatment with either PF-AES or early-generation DES. In this trial, in patients with diabetes mellitus treatment with PF-AES seemed to lower the incidence of the device-oriented composite endpoint to a similar level as patients without diabetes mellitus [22]. Data from randomized comparisons of PF-AES to new-generation DES are only available from two trials. The ReCr8 trial, an all-comer non-inferiority trial, assessed clinical outcome of patients treated with PF-AES or new-generation DP-ZES. Regarding the device-oriented endpoint non-inferiority was met as no meaningful differences were observed at 12 moths. Furthermore, there were no significant differences regarding the predefined subgroup of patients with diabetes mellitus [27]. In the smaller RESERVOIR trial, 112 patients with diabetes mellitus were randomized to treatment with PF-AES or benchmark new-generation DES with permanent polymer and angiographic as well as optical coherence tomography outcomes were assessed. With respect to the primary endpoint—neointimal volume obstruction—non-inferiority of PF-AES as compared to benchmark DES in patients with diabetes mellitus was met. As expected, clinical outcomes did not differ between both study groups [28].

Comparison of the results of these trials with the results of the present analysis is not feasible due to important differences regarding patient selection criteria and follow-up duration. Of note, study devices in dedicated randomized trials do not only differ in polymer characteristics but also other features like backbone architecture or antiproliferative drugs. For that reason, neither superiority of one device over another could undeniably be attributed to their respective polymer nor do comparable outcomes necessarily lead to rejection of the hypothesis that polymer in fact makes a difference. With respect to the present trial, the absence of significant clinical outcome differences warrants the conclusions that the effect of the coating concept alone is either non-existent or below the detection limit determined by the trial design, while both study devices represent reasonable treatment options for both patients with and patients without diabetes mellitus. Future, specifically dedicated trials are warranted to further investigate the hypothesis that tailored stent design has the potential to be part of the integrative approach to cardiovascular disease in patients with diabetes mellitus. In this context, results of the ongoing SUGAR trial are therefore eagerly awaited [29].

The observation of a higher incidence of clinical events out to 10 years after percutaneous coronary intervention in patients with diabetes mellitus as compared to patients without diabetes mellitus as well as the constant accrual of events over time underlines the high cardiovascular risk patients suffering from this frequent metabolic disorder are exposed to. Continued efforts to improve prevention and treatment of diabetes mellitus are, therefore, of ongoing importance.

### Limitations

Our study has several limitations. Although this analysis is the first to report clinical follow-up out to 10 years after treatment with PF-SES or DP-ZES, the trial was not specifically powered for a comparison of clinical outcomes in the subgroup of patients with or without diabetes mellitus. The present analysis is a post hoc analysis and, therefore, vulnerable to all methodical flaws inherent to post hoc analysis of such kind of subgroups and the respective findings need to be interpreted against this background. Furthermore, while long-term follow-up is an important strength of the present analysis, with longer follow-up duration diabetes status will change in some cases and potentially dilute findings regarding the comparison of patients with vs. without diabetes mellitus. Interestingly however, during 10-year follow-up less than 5% of patients were newly diagnosed with diabetes mellitus.

## Conclusion

At 10 years, both new-generation DES show comparable clinical outcome irrespective of diabetic status or polymer strategy. Event rates after PCI in patients with diabetes mellitus are considerable higher than in patients without diabetes mellitus and continue to accrue over time.

## Supplementary Information

Below is the link to the electronic supplementary material.

Supplementary file1 (DOCX 32 kb)

## Data Availability

The datasets on which the conclusions of the manuscript are based are presently not deposited in a publicly accessible repository as the ethics committee approval from the Technische Universität München (2007) did not foresee provision for this.

## Abbreviations

CAD: Coronary artery disease
PCI: Percutaneous coronary intervention
DES: Drug-eluting stent
PF-SES: Polymer-free sirolimus- and probucol-eluting stent
DP-ZES: Durable polymer zotarolimus-eluting stent
MACE: Major adverse cardiac events
PF-AES: Polymer-free amphilimus-eluting stent
PF-BES: Polymer-free biolimus-eluting stent
