# Supplementary material for: Ten-year clinical outcomes of polymer-free versus durable polymer new-generation drug-eluting stent in patients with coronary artery disease with and without diabetes mellitus: Results of the Intracoronary Stenting and Angiographic Results: Test Efficacy of Sirolimus- and Probucol- and Zotarolimus-Eluting Stents (ISAR-TEST 5) trial
Source: Clin Res Cardiol. 2021 Jun 22;110(10):1586–98. doi: 10.1007/s00392-021-01854-7 (PMC8484170; doi:10.1007/s00392-021-01854-7)
Supplement: Supplementary file 1 — Supplementary file1 (DOCX 32 kb) [file 392_2021_1854_MOESM1_ESM.docx]

Supplemental Table 1. Baseline Patient and Lesion Characteristics according to diabetic status

| **Characteristics** | **Patients with diabetes mellitus** | **Patients without diabetes mellitus** | ***P*** |
| --- | --- | --- | --- |
| **Patients** | N=870 | N=2132 |  |
| Age, y, ±SD | 68.5 (±10.0) | 67.5 (±11.4) | 0.02 |
| Male sex | 641 (73.7) | 1654 (77.6) | 0.03 |
| Insulin dependent diabetes | 306 (35.2) |  |  |
| Oral antidiabetic medication | 438 (50.3) |  |  |
| Arterial hypertension | 637 (73.2) | 1365 (64.0) | <0.001 |
| Current smoker | 157 (18.0) | 366 (17.2) | 0.60 |
| Hyperlipidemia | 577 (66.3) | 1330 (62.4) | 0.05 |
| Coronary artery disease |  |  | <0.001 |
| 1-vessel disease | 90 (10.3) | 399 (18.7) |  |
| 2-vessel disease | 189 (21.7) | 582 (27.3) |  |
| 3-vessel disease | 591 (67.9) | 1151 (54.0) |  |
| Clinical presentation |  |  | 0.002 |
| Instable Angina | 159 (18.3) | 406 (19.0) |  |
| Non-ST-segment elevation acute coronary syndrome | 118 (13.6) | 238 (11.2) |  |
| Silent Ischemia | 51 (5.9) | 152 (7.1) |  |
| Stable angina | 478 (54.9) | 1089 (51.1) |  |
| ST-segment elevation myocardial infarction | 64 (7.4) | 247 (11.6) |  |
| Prior myocardial infarction | 262 (30.1) | 623 (29.2) | 0.66 |
| Prior coronary artery bypass grafting | 93 (10.7) | 191 (9.0) | 0.16 |
| Body Mass Index, ±SD | 29.2 (±4.9) | 27.1 (±4.3) | <0.001 |
| Ejection fraction, %, ±SD | 51.0 (±12.4) | 53.1 (±11.4) | <0.001 |
| **Lesions** |  |  |  |
| Vessel |  |  | 0.09 |
| LAD | 366 (42.1) | 985 (46.2) |  |
| LCx | 239 (27.5) | 523 (24.5) |  |
| RCA | 265 (30.5) | 624 (29.3) |  |
| Ostial | 144 (16.6) | 389 (18.2) | 0.29 |
| Bifurcational | 175 (20.1) | 526 (24.7) | 0.01 |
| Chronic occlusion | 53 (6.1) | 113 (5.3) | 0.44 |
| Data are shown as number (Kaplan–Meier estimates as percentages) or mean (± SD) | | | |

## **Supplemental Table 2.** Clinical Outcomes at 10 years according to diabetic status

|  | **With Diabetes**  **N=870** | **HR**  **(95% CI)** | **Without Diabetes**  **N=1427** | ***P*** |
| --- | --- | --- | --- | --- |
| **MACE** | 628 (76.4) | 1.47 (1.34 – 1.62) | 1275 (62.4) | <0.001 |
| All cause death | 363 (46.6) | 1.73 (1.52 – 1.96) | 617 (31.5) | <0.001 |
| Any myocardial infarction | 91 (10.4) | 1.86 (1.35 – 2.56) | 64 (7.3) | <0.001 |
| Any Revascularization | 406 (54.6) | 1.38 (1.23 – 1.56) | 823 (42.5) | <0.001 |
| Cardiac death | 254 (37.1) | 1.92 (1.64 – 2.23) | 401 (22.8) | <0.001 |
| Target vessel related myocardial infarction | 46 (5.7) | 1.87 (1.28 - 2.74) | 64 (3.3) | 0.001 |
| TLR | 192 (28.0) | 1.53 (1.28 – 1.82) | 354 (19.2) | <0.001 |
| Data are shown as number (Kaplan–Meier estimates as percentages), hazard ratios are derived from Cox proportional hazard models, and P values are derived from Cox proportional hazard models. | | | | |

## **Supplemental Table 3.** Stent Thrombosis at 10 years according to diabetic status

| **Event** | **Patients with diabetes**  **N=870** | **Hazard ratio**  **(95% CI)** | **Patients without diabetes mellitus**  **N=2132** | ***P*** |
| --- | --- | --- | --- | --- |
| Definite stent thrombosis | 11 (1.3) | 2.63 (1.14-6.06) | 11 (0.6) | 0.02 |
| Probable stent thrombosis | 10 (1.2) | 1.82 (0.81-4.11) | 14 (0.7) | 0.15 |
| Definite/probable stent thrombosis | 21 (2.5) | 2.18 (1.22-3.90) | 25 (1.3) | 0.01 |
| Data are shown as number (Kaplan–Meier estimates as percentages), hazard ratios are derived from Cox proportional hazard models, and P values are derived from Cox proportional hazard models. | | | | |
